# Layer-By-Layer Fabrication of Large and Thick Human Cardiac Muscle Patch Constructs With Superior Electrophysiological Properties

**DOI:** 10.3389/fcell.2021.670504

**Published:** 2021-04-16

**Authors:** Danielle Pretorius, Asher M. Kahn-Krell, Xi Lou, Vladimir G. Fast, Joel L. Berry, Timothy J. Kamp, Jianyi Zhang

**Affiliations:** ^1^Department of Biomedical Engineering, School of Medicine and School of Engineering, University of Alabama at Birmingham, Birmingham, AL, United States; ^2^Department of Cell and Regenerative Biology, University of Wisconsin–Madison, Madison, WI, United States

**Keywords:** hearts, tissue engineering, layer-by-layer fabrication, stem cell, superior electrophysiology

## Abstract

Engineered cardiac tissues fabricated from human induced pluripotent stem cells (hiPSCs) show promise for ameliorating damage from myocardial infarction, while also restoring function to the damaged left ventricular (LV) myocardium. For these constructs to reach their clinical potential, they need to be of a clinically relevant volume and thickness, and capable of generating synchronous and forceful contraction to assist the pumping action of the recipient heart. Design prerequisites include a structure thickness sufficient to produce a beneficial contractile force, prevascularization to overcome diffusion limitations and sufficient structural development to allow for maximal cell communication. Previous attempts to meet these prerequisites have been hindered by lack of oxygen and nutrient transport due to diffusion limits (100–200 μm) resulting in necrosis. This study employs a layer-by-layer (LbL) fabrication method to produce cardiac tissue constructs that meet these design prerequisites and mimic normal myocardium in form and function. Thick (>2 mm) cardiac tissues created from hiPSC-derived cardiomyocytes, -endothelial cells (ECs) and -fibroblasts (FBs) were assessed, *in vitro*, over a 4-week period for viability (<6% necrotic cells), cell morphology and functionality. Functional performance assessment showed enhanced *t*-tubule network development, gap junction communication as well as previously unseen, physiologically relevant conduction velocities (CVs) (>30 cm/s). These results demonstrate that LbL fabrication can be utilized successfully to create prevascularized, functional cardiac tissue constructs from hiPSCs for potential therapeutic applications.

## Introduction

The use of engineered cardiac muscle patch constructs focusing on repair and functional restoration following an acute myocardial infarction (AMI), have shown promise in improving clinical outcomes for patients (Lake et al., 1985; Lucas and Szweda, 1999; Murphy and Steenbergen, 2008). Clinically, AMI is associated with postinfarction left ventricular (LV) remodeling and heart failure (Henkel et al., 2006; Gorenek et al., 2014). Cell therapy approaches utilizing products associated with human induced pluripotent stem cells (hiPSCs) have been examined pre-clinically as a viable therapeutic option (Xiaojun et al., 2012, 2014; Ye et al., 2014). Despite these promising results, the search continues for an effective method of restoring both mechanical and electrical function to the AMI damaged region of the heart, which is necessary for a successful therapeutic application. Furthermore, electrical function is largely dependent upon the electrical excitability of the cells and subsequently the engineered tissue. Unfortunately, due to their immature phenotype, hiPSC-derived cardiomyocytes (iCMs) tend to have reduced electrical excitability compared to mature, adult CMs found in the native myocardium (O’Hara et al., 2011; Mummery et al., 2012).

One of the key indicators for CM functional maturity at the tissue level is the capacity to support fast action potential (AP) conduction. In cardiac tissue, AP represents the time-dependent changes in the transmembrane electrical potential in CMs, which occur during each heartbeat, and are functions of highly coordinated, time- and voltage-dependent changes in activity of various ion channels and transporters (Liu et al., 2016). Conduction velocities (CVs) of genetically purified iCMs in monolayer format have been reported as high as ∼21 cm/s (Lee et al., 2012), with more complex 3D systems still lagging behind at reported CVs around 14–17 cm/s (Gao et al., 2018; Pretorius et al., 2020) with the potential of reaching up to 25 cm/s when highly organized (Gao et al., 2017). However, promising, the CVs reported for hiPSC-derived tissue still fall short of those observed in native adult LV tissue (∼30–100 cm/s; Valderrábano, 2007; Yang et al., 2014). Some of the highest CVs reported for engineered cardiac tissue have been obtained with neonatal rat CMs, and was recorded at 32.3 ± 1.8cm/s, yet at their thickest, these structures were recorded to only be 100 μm (Jackman et al., 2018).

Utilization of promising fabrication strategies which yield highly organized structures, such as the layer-by-layer (LbL) approach, allow for superior control over a range of physicochemical properties. In a previous study, we demonstrated that our LbL fabrication method yields thick, vascularized engineered cardiac tissue, with CVs ranging between 17 and 19 cm/s, as well as viscoelastic properties that were similar to native murine myocardial tissue (Pretorius et al., 2020), yet this study only utilized CMs and endothelial cells (ECs). It is known, however, that fibroblasts (FBs) play key roles in the healthy native adult mammalian heart. FBs contribute to general heart function, homeostasis, and structure, most notably during the production and remodeling of extracellular matrix (ECM). Culturing CMs in the presence of FBs has also been shown to affect the electrophysiological properties of the CMs (Zhang et al., 2019).

In the present study, we aimed at developing a LbL approach to engineer large and thick tri-lineage (CM, EC, and FB) cardiac tissue constructs. The engineered cardiac tissue was characterized at various time points, over a 4 week period, in order to qualify and quantify the changes that occur in the structures *in vitro*, which ultimately lead to a better potential understanding of the remodeling that these structures undergo *in vivo*. We hypothesized that by mimicking the cellular composition of the native myocardium, i.e., utilizing CMs, ECs, and FBs, with a LbL fabrication process would not only allow for the production of thicker tissues but also increase their ability to mimic the native myocardium more closely in terms of form and function. Characterization included (1) histology and immunostaining to analyze the cellular and structural morphology, (2) immunostaining to quantify viability, (3) immunostaining of 2D cryosections and 3D structures to analyze cell migration, potential vascularization, and final cellular fate, (4) immunostaining and quantification of ECM remodeling, (5) RNA analysis to quantify and corroborate the results obtained from immunostaining, and (6) optical mapping to determine the AP and CV to assess functionality.

## Materials and Methods

### Cell Culture and Characterization

Human cardiac fibroblast induced pluripotent stem cells were reprogrammed from human cardiac FBs, and subsequently differentiated into hiPSC-cardiomyocytes (iCMs), as previously reported (Lian et al., 2012; Burridge et al., 2014; Ye et al., 2014). Generally, spontaneous iCM contractions were observed between days 7 and 10 after commencement of the differentiation protocol, with beating numbers increasing up to day 12. Metabolic purification of iCMs was achieved via glucose deprivation (RPMI 1640 without glucose, supplemented with sodium DL-lactate and B27^+^, Gibco) for 3–6 days, initiated at day 9, allowed for a population of iCMs that yielded a minimum of 95% cTnT positive. hiPSCs were maintained at optimal conditions, as previously described (Zhu et al., 2017), on six-well plates coated with Matrigel (Corning), using mTeSR 1 maintenance media (STEMCell Technologies, Canada).

Human induced pluripotent stem cell-endothelial cells (iECs) were differentiated as described previously (Zhang et al., 2014; Gao et al., 2018; Su et al., 2018). Briefly, undifferentiated hiPSCs were seeded into 0.5 mL fibrin scaffolds and treated with CHIR99021 and U46619 in EBM2 medium (Lonza, United States) supplemented with B27^–^ for 24 h. The medium was then replaced with EBM2 which was supplemented with B27^–^, vascular endothelial growth factor (VEGF), erythropoietin (EPO), and transforming growth factor β1 (TGFβ1), and then cultured for an additional 96 h with a media change midway through. Finally, the scaffolds were released and cultured in EGM2-MV medium (Lonza, United States) supplemented with B27^+^, VEGF, and SB-431542, with media changes every 2 days. hiPSC-ECs were purified and enriched via collection of cells positive for CD31 using fluorescence-activated cell sorting (FACSAria II). Antibodies used for selection and cell characterization are listed Supplementary Table 1. See Supplementary Figure 1 for cell characterization and Supplementary Figure 2 for the proliferation assay showing the lack of tumorigenic properties of the iCMs utilized in this study.

Human induced pluripotent stem cell-FB (cFB) differentiation commenced using hiPSC-line DF19-9-11T with cells at 100% confluence (day 0) (Zhang et al., 2019). The hiPSC culture medium was changed to RPMI/B27^–^ and supplemented with 12 μM CHIR99021, with cells treated in this medium for 24 h (day 1). Medium was changed once again to RPMI/B27^–^ for another 24 h (day 2). Within 24 h (before day 3), the medium was changed to cardiac FB differentiation basal medium (CFBM, see Supplementary Table 2) supplemented with 75 ng/mL bFGF (WiCell Research Institute). Cells were then cultured with CFBM + 75 ng/mL bFGF every other day up to day 20 when they were utilized for flow cytometry analysis and passaged for subsequent use in tissue fabrication.

### Fibrin Matrix Composition

The fibrin matrix used (per milliliter) used for the differentiation of the iECs as well as for the construction of each alternating layer of the cardiac tissue during the modular fabrication process consisted of the following components as was defined previously (Gao et al., 2018): 0.12 mL fibrinogen (25 mg/mL, Sigma-Aldrich, CAS# 9001-32-5), 0.02 mL Matrigel (Corning, # 356235), 0.56 mL of HEPES (20 mM, pH 7.4, Corning), 0.001 mL CaCl_2_ (2 M), 0.3 mL DMEM (Gibco, High glucose, # 11965-118), and 0.006 mL thrombin (80 U/mL, MP Biomedicals).

### Polydimethylsiloxane Platforms

Polydimethylsiloxane (PDMS) platforms were fabricated by mixing PDMS (Dow Corning Sylgard 184 Silicone, Product #2065622) in a 10:1 elastomer:curing agent ratio and poured into a 100 mm diameter Pyrex Petri dish (Corning, # 3160102). These were cured at 75°C for 2 h in an oven, after which custom platforms of 10 mm × 5 mm (2.5 mm thick) were cut. Prior to their use in the tissue fabrication and culture process, all PDMS structures were autoclaved.

### Optimized LbL Engineered Tissue Fabrication

**FIGURE 1:**
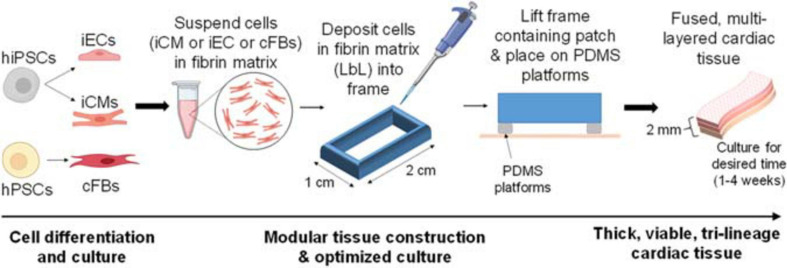
Basic description of optimized cardiac tissue surrogate fabrication process, allowing for extended culturing of thick tissue structures. The first layer of cells is deposited and cultured for 48 h, after which the second layer is deposited using layer-by-layer (LbL) approach. The second layer of cells is deposited, allowed to polymerize, and the entire structure contained in its frame is then lifted off of the dish surface and placed on top of Polydimethylsiloxane (PDMS) platforms. The third layer of cells is deposited in a similar fashion. Thick, viable, multi-layered tissue can be cultured for the desired amount of time (1–4 weeks).

Following cell differentiation, cardiac tissue fabrication commenced (Figure 1). Petri dishes (BioLite Cell Culture Treated Dishes, Thermo Scientific) were coated with a 5% pluronic F-68 solution (Gibco, # 24040032) and incubated at 4°C overnight. The pluronic solution was removed, and a sterile polycarbonate frame (internal area: 1 × 2 cm^2^) was attached with a 2% agarose solution. Note that the frames were modified with channels to allow for maximal media contact following tissue fabrication. iCMs were dissociated (STEMdiff Cardiomyocyte Dissociation Medium, STEMCell) and mixed with the fibrin matrix at a concentration of 10 × 10^6^ cells/mL. Note that the deposition of the iCM layer denotes “D0” for the remainder of the fabrication process. 400 μL of this solution was quickly deposited into each mold to produce the first layer. Following complete polymerization, the culture medium was added (STEMdiff Cardiomyocyte Support Medium, 2 mg/mL ε-aminocaproic acid) and incubated at 37°C (5% CO_2_) for 2 days. The next layer, comprised of iECs, was made in a similar fashion, with the following exceptions: iECs were dissociated using trypsin (0.25% trypsin, 0.1% EDTA, Corning, # 25053CI) and then mixed with the fibrin matrix at a concentration of 10 × 10^6^ cells/mL. 200 μL of this solution was quickly deposited into each mold, yielding a 2:1 ratio of iCMs:iECs (Gao et al., 2018). Fresh culture medium (10% fetal bovine serum, 2% B27^+^, and 2 mg/mL ε-aminocaproic acid, 10 μM ROCK inhibitor in DMEM) was added, following layer polymerization. After 24 h, the frame containing the engineered cardiac tissue was lifted off of the dish surface and placed on top of custom-cut PDMS stilts, allowing for the tissue to be fully suspended in fresh culture media (2% fetal bovine serum, 2% B27^+^, and 2 mg/mL ε-aminocaproic acid in DMEM). After a further 48 h, the next layer, comprised of cFBs, was made in a similar fashion, with the following exceptions: cFBs were dissociated using TrypLE (Gibco, # 12604013) and then mixed with the fibrin matrix at a concentration of 10 × 10^6^ cells/mL. 100 μL of this solution was quickly deposited into each mold, yielding a 2:1:0.5 ratio of iCMs:iECs:cFBs (Pinto et al., 2016; Gao et al., 2018). Incubation continued for the desired period of time (1–4 weeks in total), with media replacement once per week.

### Tissue Preservation

Fabricated tissue samples were fixed in 4% formaldehyde (Pierce, Thermo Scientific, # 28906) for 1 h prior to embedding in either optimal cutting temperature compound (OCT compound, Fisher Health Care, United States) or paraffin for histological analysis. Histological analyses were performed on 10 μm sections. Whole-mount samples were stored in PBS until staining.

### Histochemistry

Deparaffinized and rehydrated sections were stained with hematoxylin solution (Mayer’s, Merck, 3 min) followed by working eosin Y solution (2 min). Following subsequent dehydration, all samples were mounted with Permount and imaged using a bright field microscope (Olympus IX83 epifluorescent microscope).

### Immunohistochemistry

Fixed OCT embedded sections were blocked and permeabilized for 30 min in 10% donkey serum, 3% BSA, and 0.05% Triton-X. Following addition of primary antibodies, samples were incubated for 1 h at room temperature (Supplementary Table 1). Samples were washed in PBS (3 × 5 min), and subsequently labeled with secondary antibodies with fluorescent tags and 4′, 6-diamidino-2-phenylindole (DAPI, 100 ng/mL) and incubated for at room temperature for 1 h (Supplementary Table 1). Sections were mounted with VECTASHIELD Antifade Mounting Medium and visualized by confocal laser scanning (Olympus FV3000 confocal microscope).

### Whole-Mount Staining

Fixed whole-mount samples were blocked and permeabilized in 10% donkey serum, 10% Tween-20, 3% BSA, 0.05% Triton-X, and 0.02% sodium azide in PBS overnight at 4°C. Then, primary antibodies were added and samples were incubated overnight at 4°C (Supplementary Table 1). Samples were washed in PBST (3 × 10 min) and fluorescently labeled secondary antibodies along with DAPI were added followed by another overnight incubation at 4°C. Following washing, tissue was cleared according to the previously described protocol (Li et al., 2019) with 3 h incubation in Ce3D clearing agent. Whole-mount constructs were transferred to an Ibidi μ-Slide (# 80286), covered with VECTASHIELD Antifade Mounting Medium, and visualized via confocal laser scanning (Olympus FV3000 confocal microscope).

### RNA Isolation and Analyses

Samples for RNA analysis were suspended in TRIZOL (Invitrogen) and homogenized. RNA extraction was completed using Direct-zolRNA MiniPrep Plus (Zymo Research Corporation) according to the manufacturer’s protocol. Following concentration measurement on the Nanodrop device, 1 μg of RNA was converted into complementary DNA (cDNA) through the reverse transcription reaction using the SuperScript IV VILO Master Mix (Thermo Fisher Scientific) then diluted to a final concentration of 5 ng/μL. qPCR analysis of each sample was performed on a QuantStudio 3 real-time PCR system using PowerUp SYBR Green Master Mix (Thermo Fisher Scientific). Quantification of relative expression was done by normalizing to sample GAPDH expression. Primers used for RT-qPCR analysis are included in Supplementary Table 3.

### Optical Mapping

To assess action potential duration (APD), CV, and minimum pacing cycle length, engineered LbL tissue samples were stained with a voltage-sensitive dye RH-237 (2.5 μM) for 10 min and transferred to a perfusion chamber mounted on an inverted microscope. Control samples consisted of iCM:iEC bilayered tissue, while test samples consisted of iCM:iEC:cFBs. Samples were constantly perfused with Hank’s balanced salt solution (HBSS) at approximately 37°C. Pacing/stimulation of all samples was done with a bipolar electrode consisting of a glass pipette filled with HBSS and a silver wire coiled around its tip. A micromanipulator was used to position the electrode tip at the sample’s edge. Rectangular stimulation pulses, with a duration of 2 ms and current strength 1.5-times the excitation threshold were used. Fluorescence was excited with a 200-W Hg/Xe arc lamp and recorded with a 16 × 16 photodiode array (Hamamatsu) at a spatial resolution of 110 μm per diode as previously described (Sowell and Fast, 2012). Excitation light was filtered at 532–587 nm, and emitted fluorescent light was filtered at >650 nm. The perfusion solution was supplemented with 5 μM of blebbistatin to eliminate potential motion artifacts caused by the samples’ spontaneous contractions. Isochronal maps of the activation spread were constructed from activation times measured at 50% of the maximum AP amplitude. CV was calculated at each recording site from local activation times and averaged across the whole mapping area. AP duration was measured at 50 and 80% of signal recovery (APD_50_) and (APD_80_), respectively.

### Statistical Analyses

All results are reported as mean ± standard error (mean ± SEM). Significant differences between two mean values were determined via the Student’s two-tailed *t*-test; and ANOVA or repeated ANOVA with the Tukey post hoc test were used for multiple (more than two groups) comparisons or repeated measurements. *p*-values of less than 0.05 were considered statistically significant. These analyses were performed utilizing GraphPad Prism8 data analysis software package.

## Results

### Layer-By-Layer Fabrication Produces Thick, Synchronously Beating, Fused Engineered Cardiac Tissue

**FIGURE 2:**
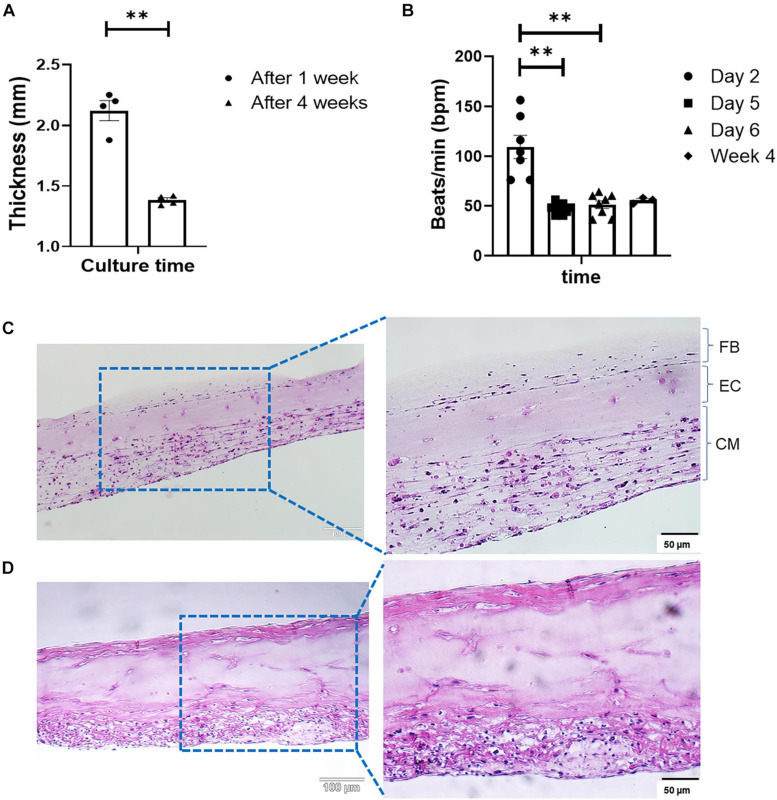
**(A)** Change in tissue thickness between week 1 and week 4, measured with a caliper (Mitutoyo 500-196-30, Digimatic, *n* = 4, **p < 0.005). **(B)** LbL engineered tissue beat-rate over the first 6 days of culture (*n* = 8) as well as week 4 (*n* = 3), **p < 0.005. H&E staining of LbL engineered tissue at **(C)** Day 7, and **(D)** Day 28 of culturing.

Engineered cardiac tissue produced with the optimized method described in Figure 1 yielded structures of 2.12 ± 0.083 and 1.38 ± 0.019 mm in thickness after 1 and 4 weeks in culture, respectively (Figure 2A, *n* = 4). The statistically significant (*p* < 0.005) reduction in tissue thickness between week 1 and week 4 are most likely due to compaction caused by the contractile forces exerted by both the iCMs as well as the cFBs. Following the deposition of the iCMs, synchronization of the iCM layer in all samples was noted after 48 h (on Day 2). The resulting beating-rate (per min) of each engineered tissue was determined (Figure 2B), yielding a rate of 109 ± 11.5 beats/min (bpm). Following the iEC layer addition on Day 3 and an additional 2 days of culture (Day 5), the beating-rate on the fifth day of culture, prior to cFB deposition had decreased significantly, to 47 ± 2.0 bpm. Following the deposition of the cFBs, and an additional 24 h of culture (Day 6), the beating-rate of the tissues was noted at 51 ± 3.9 bpm. After 4 weeks in culture, the beating-rate of the constructs were noted at 55 ± 1.8 bpm.

Modification to the ECM composition between weeks 1 and 4 were observed via H&E staining (Figures 2C,D). Histology shows increases in structural ECM components such as collagen (increased intensity as well as the distribution of eosinophilic staining, Figures 2C,D). As sample time in culture increased, compaction of the structures the degree of compaction increased (Figure 2D), and structures, although initially somewhat disorganized (Figure 2C), began resembling *in vivo* muscle. The alterations in the ECM composition, specifically those associated with collagen deposition were attributed to both the culture of iCMs as well as the addition of cFBs, based on the strong eosinophilic staining noted in both the bottom as well as the top layers of the structure at week 4 (Figure 2D). Fusion and coupling of the structure layers was confirmed by not only the synchronous macroscopic beating of the engineered tissue (Figure 2B) but also via H&E staining. Another observation made via H&E was that of cell migration over the culture period. As with a previous study by this group (Pretorius et al., 2020), it was noted that iECs initiated migration and re-arrangement out of their originally deposited layer into the resident iCM layer within less than 48 h post-deposition, as is clear from the somewhat acellular appearance of the middle layer of the engineered tissue (Figure 2C). The degree of cell migration and potential iEC re-arrangement was confirmed with cell-specific immunofluorescent markers (Figure 4A). It should be noted that structures visualized here via H&E staining appear thinner than those preserved in OCT or when measured with a caliper. This difference in overall tissue thickness is attributed to the multiple dehydration-related steps required during the H&E processing of the hydrogel structures leading to decreased preservation of the original architecture.

### Layer-By-Layer Fabrication Results in Engineered Tissue With Limited Necrosis Markers

**FIGURE 3:**
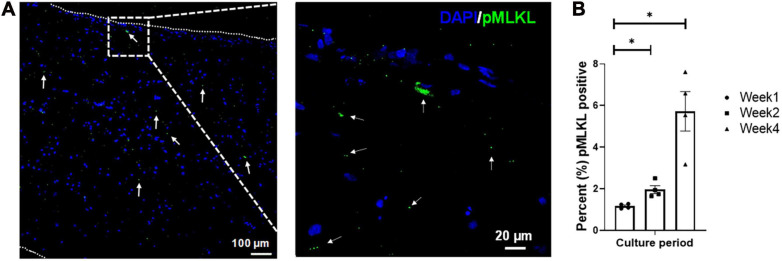
Representative confocal micrograph of engineered LbL cardiac tissue displaying necrotic cells identified by the necrosis marker phosphorylated MLKL (Ser358, pMLKL) at **(A)** week 1 of culture, with **(B)** the percentage of necrotic cells in LbL engineered cardiac tissue (*n* = 4, *p < 0.05). Border indicated with short dashed line.

Tissue viability was determined via pMLKL staining (Figure 3), which specifically stains for cell necroptosis (Linkermann et al., 2014) and is associated with inflammatory markers (Negroni et al., 2017). The pMLKL-positive cells were quantified as a percentage (%) of the total number of cells present (i.e., normalized to the DAPI staining, *n* = 4 with a minimum of five images taken per sample). The initial degree of necrosis noted in the engineered tissues was very low, and quantified at 1.17 ± 0.06%. After an additional week of *in vitro* culture, the degree of necrosis increased to 1.96 ± 0.19%, while the degree of necrosis after 4 weeks in culture was observed to be 5.73 ± 0.95%. Even though the percentage of pMLKL positive cells increased significantly from week 1 to week 2 and week 4, respectively, it still compared well with previously published data from our group, showing 5.6 ± 1.4% after 4 weeks in culture of engineered structures that were initially ∼1.73 mm thick at week 1 (Pretorius et al., 2020).

### Cellular Migration and Fate

**FIGURE 4:**
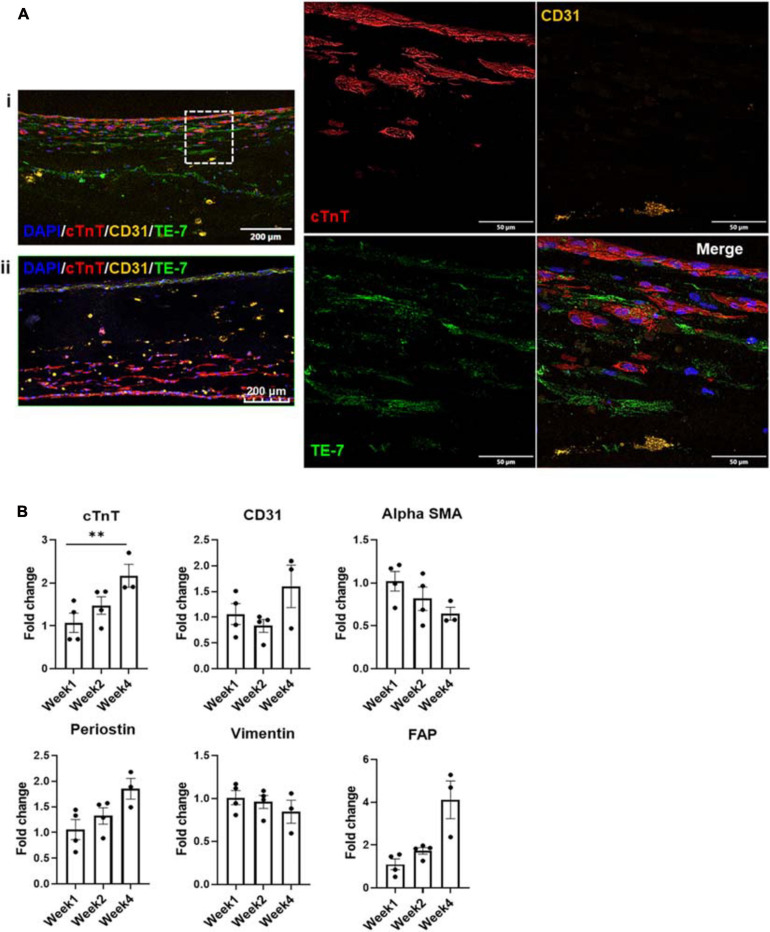
**(A)** Fluorescent staining with cTnT, CD31, and TE-7 showing tri-lineage engineered tissue after (i) one and (ii) 4 weeks in culture, respectively, along with **(B)** expression levels of cell-specific markers at weeks one, two, and four, respectively (*n* = 4, *p < 0.05, **p < 0.005).

Imaging of the fluorescently stained sections (Figure 4Ai) showed migration of the iECs as well as the cFBs toward their iCM counterparts within a number of days after these layers had been deposited. The migration process continued over the 4 weeks of culture, yet leaving no area of the engineered structure completely acellular (Figure 4Aii).

**FIGURE 5:**
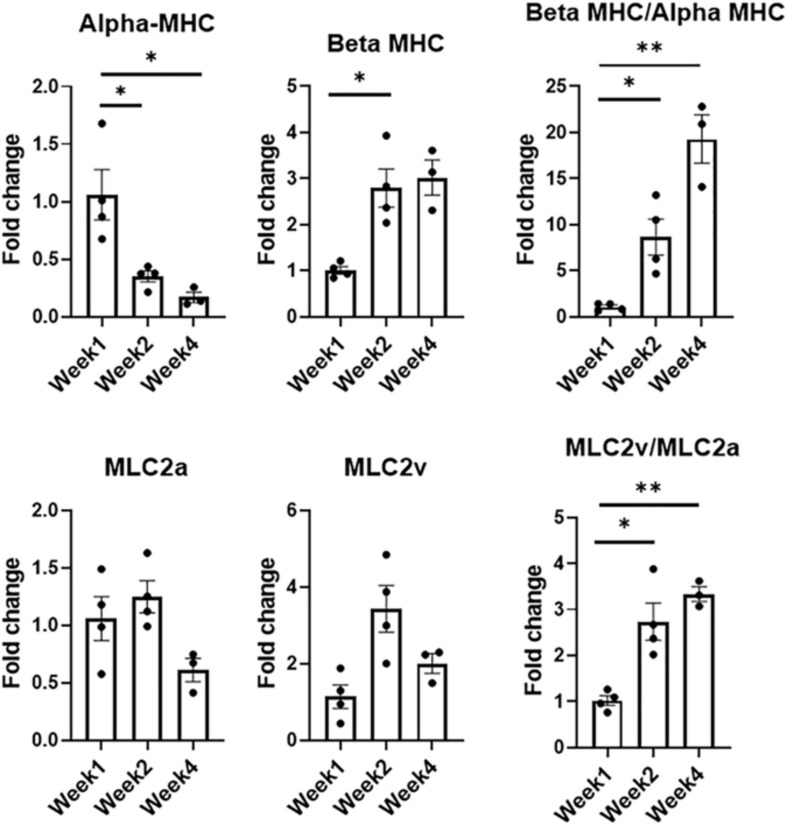
Expression levels of CM-specific maturation and phenotypic markers at weeks one, two, and four, respectively (*n* = 4 for week 1 and week 2, *n* = 3 for week 4, *p < 0.05, **p < 0.005).

Expression levels of various cell markers were monitored over the culture period, i.e., cTnT for iCMs, CD31 for iECs as well as α-SMA (alpha smooth muscle actin), periostin, vimentin and FAP (FB activation protein) for cFBs (Figure 4B). At each time point, at least three tissue samples were used to determine the respective expression levels. No statistically significant differences were noted in the expression levels of CD31, α-SMA, periostin, and vimentin or FAP, suggesting that the expression levels of these factors were stable throughout the culturing period. The slight decrease noted in vimentin expression can potentially be attributed to senescence of the cFB line, which is known to occur *in vitro* after a specific number of cell divisions and/or passages (Zhang et al., 2019). The slight increases in factors such as periostin, rather, could potentially be suggestive of an environment that is conducive to cellular motility (Gillan et al., 2002). The only statistically significant difference was noted in the expression level of cTnT when comparing week 1 to week 4. This increase could more likely than not be attributed to the maturation of the iCMs in the engineered tissue, since iCMs are terminally differentiated cells and minimal proliferation is observed in these cell populations (see Supplementary Figure 2). To further investigate the potential maturation of the iCMs in the engineered tissues, the expression levels of known maturation markers as well as chamber-specific markers were examined (Figure 5).

Developmental and hormonal factors control the expression of alpha- and beta-myosin heavy chain (MHC), two functionally distinct, species-dependent cardiac MHC isoforms (Everett et al., 1984; Allen and Leinwand, 2001). Not only have these isoforms been shown to be correlated with contractile capabilities, but alterations in expression levels are also age-, and species-dependent (van der Velden et al., 1998). Switches in expression from one isoform to another have been highly referenced in literature in developing systems, with increases in β-MHC and more specifically β-MHC:αMHC, being a key indicator of CM maturation (Reiser et al., 2001; Mihic et al., 2014; Yang et al., 2014; Ruan et al., 2015; LaBarge et al., 2019). During the culturing period, there was a statistically significant decrease in the expression levels of alpha-MHC (α-MHC), noted when comparing the expression levels of week 1 and week 2 as well as week 1 and week 4, respectively. Week 2 also saw an almost threefold increase (*p* < 0.05) in the expression level of beta-MHC (β-MHC) when compared to week 1. Analysis of this ratio showed an eightfold increase from week 1 to week 2 (*p* < 0.05), and a 19-fold increase from week 1 to week 4 (*p* < 0.005).

Expression levels for chamber-specific markers, i.e., atrial (MLC2a) and ventricular (MLC2v), were also compared (Ng et al., 2010). Analysis showed no statistically significant differences in MLC2a or MLC2v between any of the time points. Interestingly enough, when comparing the expression levels of ventricular:atrial markers, i.e., MLC2v:MLC2a, a 2.7-fold increase (*p* < 0.05) was noted between week 1 and week 2, while a 3.3-fold increase (*p* < 0.005) was observed between week 1 and week 4. With MI affecting the LV more prominently, having cells with a more ventricular-like phenotype present in the engineered tissues is highly advantageous.

### Dynamic ECM Evolution

**FIGURE 6:**
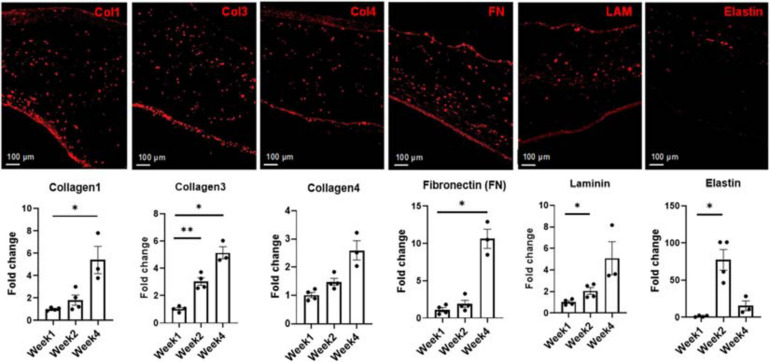
Representative images and subsequent quantification of ECM evolution over 4 weeks in culture, showing the deposition of Collagen 1, Collagen 3, Collagen 4, Fibronectin (FN), Laminin (LAM), and Elastin at week 4. Expression levels of each extracellular matrix (ECM) marker was determined via RNA analysis. Scale bars = 100 μm, *n* = 4 for week 1 and week 2, *n* = 3 for week 4, *p < 0.05, **p < 0.005.

Throughout the 4-week culturing period, the engineered cardiac tissue structure ECM underwent remodeling to various degrees. As demonstrated in a previous study, due to fibrinolysis, the majority of the fibrin matrix degrades within the first 2 weeks (Pretorius et al., 2020) (see Supplementary Figure 3). For this study, the degree to which the remodeling occurs, and how the addition of the cFBs affected potential matrix deposition compared to our previous study, was monitored and expression levels quantified via RNA analysis. For this analysis, at least three tissue samples were used per time point. With knowledge obtained from prior studies regarding the ECM remodeling during the culturing of iCMs (Bax et al., 2012; Wendel et al., 2015; Pretorius et al., 2020), samples were again analyzed for additional ECM components, including collagen 1 (Col1), collagen 3 (Col3), collagen 4 (Col4), laminin (Lam), and fibronectin (FN) (Figure 6). Samples were also analyzed for elastin due to the incorporation of iECs and cFBs as well as the potential for vessel formation (Mecham et al., 1983; L’Heureux et al., 1998). Quantification showed a nearly fivefold increase in collagen 1 production from weeks 1 to 4. This trend compared well with our previous studies, where bi-layered structures consisting of only iCMs and iECs yielded a threefold increase in collagen 1 production (Pretorius et al., 2020). It is known that cFBs are vital in synthesizing ECM proteins, including, but not limited to, fibrillar collagen types 1 and 3, basement membrane type 4 collagen, FN, and laminin (Eghbali, 1992). Interestingly enough, the increasing trends in collagen 1 production were also associated with the upregulation trends in periostin (Figure 4B), a known proponent of collagen fibrillogenesis and ECM remodeling (Takayama et al., 2006; Norris et al., 2007). Similarly, there was an upregulation trend in the expression levels of FAP. Previous studies have shown that collagen induces FAP expression via binding of α3β1 integrin (Kennedy et al., 2009).

Statistically significant increases were observed in the expression levels of collagen 3 when comparing week 1 to week 2 (*p* < 0.005) and week 1 to week 4 (*p* < 0.05), respectively. Expression of collagen 4 levels increased at both week 2 and week 4 compared to week 1, though this increase was not statistically significant. An almost 10-fold increase in the expression level of FN was noted when comparing week 1 to week 4. Statistically significant increases in the expression levels of laminin was noted between week 1 and week 2 (*p* < 0.05). An almost 70-fold increase in the expression level of elastin was observed from week 1 to week 2, followed by a decrease at week 4. One potential cause of this sharp decrease in the elastin expression level could be linked to bFGF production by the iECs in the engineered constructs (Savchenko et al., 2019). Previous studies have shown that free bFGF decreases the expression levels of elastin at the transcriptional level in culture (Rich et al., 1996).

### Functional Performance of Tri-Lineage Engineered Tissue

**FIGURE 7:**
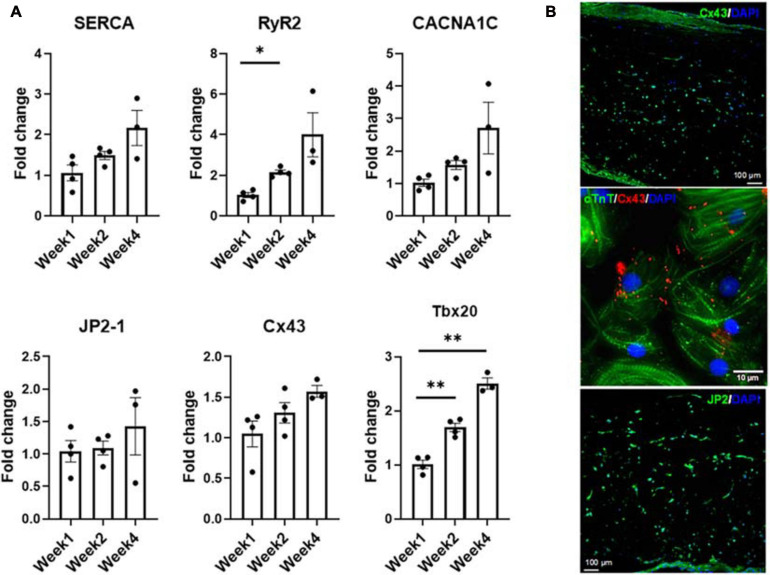
**(A)** Expression levels of CM-specific functional markers at weeks one, two, and four, respectively (*n* = 4 for week 1 and week 2, *n* = 3 for week 4, *p < 0.05, **p < 0.005). **(B)** Representative confocal micrograph of tri-lineage engineered cardiac tissue at week 2 stained for Cx43 and JP2, respectively.

**FIGURE 8:**
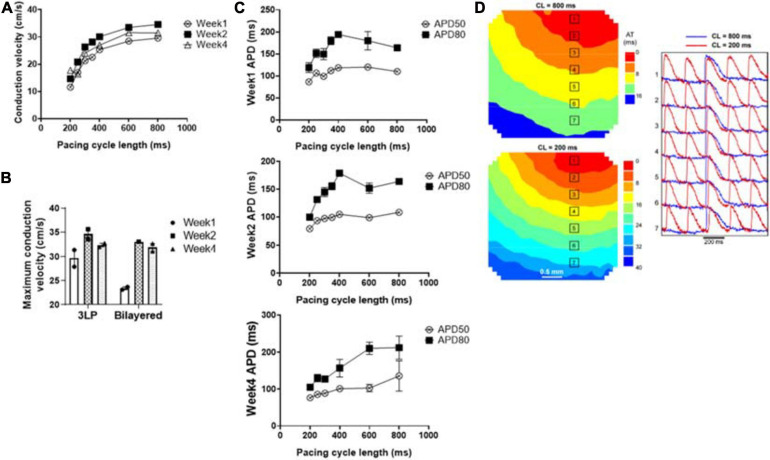
Optical mapping results showing **(A)** the average conduction velocity of the tri-lineage engineered cardiac tissue at over the 4-week culture period as a function of pacing cycle length (*n* = 4), **(B)** the maximum conduction velocity of the tri-lineage engineered cardiac tissue compared to bilayered control engineered cardiac tissue, at each time point, respectively (*n* = 4 with *p < 0.05), **(C)** APD_50_ and APD_80_ of the tri-lineage engineered cardiac tissue as a function of pacing cycle length (*n* = 4). **(D)** Representative isochronal electrical activation maps of engineered cardiac tissue, with representative optical action potentials at locations indicated on maps pacing at 200 ms (red) and 800 ms (blue) cycle lengths after 1 week in culture.

Expression levels of factors associated with functional performance were assessed by RNA analysis (Figure 7) of developmental transcription factor Tbx20, calcium handling machinery (JP2, RyR2, Cx43, SERCA, and CACNA1C) and optical mapping (Figure 8) to determine CVs through the tissue. All mapping was performed over the 4 week culture period. From week 1 to week 2, there was a twofold increase in the expression level of RyR2, suggesting that some of the calcium handling machinery, specifically some of the machinery associated with sarcoplasmic reticulum (SR)-associated calcium release, improved (Ronaldson-Bouchard et al., 2018). A significant increase in Tbx20 expression was noted, with a 1.7-fold increase and a 2.5-fold increase observed from week 1 to week 2 and week 1 to week 4, respectively.

The conduction capabilities of the tri-lineage engineered cardiac tissues were evaluated over the 4 week period and compared to bilayered samples fabricated in a similar fashion, without any FBs (Figure 8). CVs were recorded between pacing cycle lengths of 200–800 ms, and average CVs are noted at the respective pacing cycle length in Figure 8A (*n* = 4 for each time point). The average CV trends show that increased time in culture, i.e., 2 weeks vs. 1 week, and 4 weeks vs. 2 weeks, allows for enhanced conduction capabilities. This trend holds true over the entire pacing frequency range, up to 200 ms (5 Hz). Maximum CVs noted at each time point, regardless of the pacing cycle length it was obtained at, are shown in Figure 8B. The maximum CV noted for each run was 25.6 ± 2.47 and 21.8 ± 0.97 cm/s for week 1, 37.3 ± 1.65 and 36.8 ± 2.59 cm/s for week 2, and 39.8 ± 4.35 and 30.0 ± 1.36 cm/s for week 4, for the tri-lineage and bilayered cardiac tissue structures, respectively. Of particular note was that CVs as high as 49 cm/s were obtained after 4 weeks in culture (Figure 8B). The tri-lineage structures showed significant increases in CV from week 1 to week 2, as well as week 1 to week 4, with an overall increasing trend. Bilayered structures also showed a significant increase in the CV when comparing values obtained at week 1 to week 2, and week 1 to week 4, but overall maximum CVs were obtained after 2 weeks in culture. Tri-lineage engineered tissue structure APD values were obtained as functions of pacing cycle length (Figure 8C). Isochronal activation maps show uniform signal propagation across the engineered cardiac tissue after 1 week of culture at pacing cycle lengths of 200 and 800 ms (Figure 8D). Isochronal activation maps of engineered tissues paced at weeks two and four also show uniform conduction (Supplementary Figure 4). The lack of observed conduction heterogeneity or conduction blocks suggests low arrhythmic risk of the constructs.

## Discussion

In this study, a modular LbL fabrication method was developed to fabricate cardiac muscle constructs from three cardiac cell types derived from human pluripotent stem cell lines. Over a 4 week period in culture, it was shown that these large and thick cardiac tissue constructs were functionally superior to previously reported engineered structures from the perspective of their larger, thicker nature, as well as superior APD CV. This study is the first to our knowledge to demonstrate robust (1 × 2 cm^2^) hiPSC-derived engineered cardiac tissues that surpass 2 mm in thickness (∼2.12 mm), without significant apoptosis/necrosis. The physiologically relevant CVs ranging from around 25 cm/s after 1 week in culture to velocities as high as 49 cm/s after 4 weeks in culture, all lacking arrhythmogenic properties, demonstrate the significant clinical relevance of these cardiac muscle constructs. Previously, the best achievable thickness of engineered cardiac tissue constructs with this level of CV was only 100 μm (Jackman et al., 2018). Quantification of the temporal stability of the expression of cTnT, CD31, α-SMA, periostin, vimentin, and FAP suggested that all three cell populations (iCM, iEC, and cFBs) were stable. Additionally, this study also considered the temporal changes that occur during ECM remodeling in these engineered tissues, via immunofluorescent staining and RNA expression levels.

We have recently reported (Gao et al., 2018) that a novel dynamically cultured engineered human cardiac muscle patch (hCMP) demonstrated a significant advancement in the field of myocardial tissue engineering, due to its clinically relevant dimensions (2 cm 4 cm 1.2 mm) as well as contractile force generation capabilities were substantially greater than had been previously achieved (Ruan et al., 2016; Shadrin et al., 2017; Yang and Murry, 2017). However, these hCMPs were manufactured by *mixing* hiPSC-CMs, -ECs, and -SMCs into a single layer of cells. In the functional myocardium, CMs are typically found adjacent to one another as opposed next to the SMCs, FBs or ECs, and the mixing of cardiac cells in engineered cardiac tissue likely preventing the hiPSC-CMs from coalescing into a fully interconnected contractile apparatus in the past. This might partially explain why force generation measurements remained lower than in native heart tissue. In the current study with an hCMP composed of layers of hiPSC-CMs, -ECs, and -FBs sandwiched in a LbL fashion, compared to a single layer of hiPSC-CMs, the three-layered patch generated approximately twice the CV compared to the previous study, suggesting a significantly improved physiology using this LbL approach.

Cardiac FBs are known to contribute significantly to the normal ECM formation. During the 4 weeks in culture, ECM remodeling occurs. The increases in collagen 3 (Figure 6) are likely a precursor to collagen 1 in this case, as it is known to play a vital role in collagen 1 fibrillogenesis and is crucial for normal cardiovascular development (Liu et al., 1997). Fibronectin, is highly expressed in the heart during the early stages of embryogenesis and has furthermore been shown to be vital in the vasculogenesis process (George et al., 1993). It has also been suggested that increases in FN expression, as noted at week 4 could be related to the regulation of vessel formation in similar engineered cardiac tissues (Pretorius et al., 2020). Even though laminin is known to be a basal lamina protein and more closely associated with cellular differentiation, migration and adhesion, it has been suggested that manipulation of the ECM laminin content may be an effective means of inducing structural and functional changes at a cellular level by altering the cardiac titin isoform ratio (Hochman-Mendez et al., 2020a). Laminins and other proteins are connected to the cell surface by costameres, which are in turn assembled by a combination of integrins and dystroglycans, and serve as a structural and functional bridge. These costameres and their subsequent integrins contribute to signal transduction, transmitting force signals between the contractile apparatus and ECM via interactions with primarily titin and other Z-line associated structures (Peter et al., 2011). This process not only supports the mechanical integrity of the sarcolemma, contributes to mechanical signaling but also provides spatial prompts for muscle fiber organization (Hochman-Mendez et al., 2020b). The upregulation in the levels of laminin observed in the tri-lineage tissues could, thus, likely be associated with the structural and functional maturation of the iCMs, as well as the enhanced tissue compaction and alignment noted in Figures 2C,D. Increases in periostin are associated with significant increases in elastin production by cardiac-specific cell, and more specifically those cells negative for CD31 and CD45 (Kanisicak et al., 2016). Furthermore, the increased expression of elastin could likely be attributed to cellular migration and re-arrangement of the ECs into more organized structures as well (Mecham et al., 1983).

Even though the increases in junctophilin (JP2), sarco/endoplasmic reticulum Ca^2+^-ATPase (SERCA), calcium voltage-gated channel subunit alpha1 C (aka Ca_*v*_1.2, encoded by CACNA1C) and connexin 43 (Cx43) were not statistically significant, there were clear trends hinting at the development of the calcium handling machinery in the tri-lineage tissues (Figure 7). The multi-chambered mammalian heart has its origin from a simple tubual structure via polar elongation, myocardial differentiation and morphogenesis. The large family of T-box (Tbx) transcription factors have been shown to be vital in their role as distinct subprograms during cardiac regionalization (Singh et al., 2005; Stennard et al., 2005). Tbx20 plays a central role in these pathways, and has important activities in both cardiac development and adult function, specifically affecting chamber differentiation and overexpression of Tbx20 has been shown to lead to increased expression of Cx43, and subsequent increases in CVs *in vivo* (Chakraborty and Yutzey, 2012). The expression of Tbx20 has also been associated with the activation of and maturation-associated genes in iCMs such as those recorded in Figure 5 (Zhou et al., 2020). The tri-lineage LbL cardiac engineered tissues fabricated here are the first, to our knowledge, to be fabricated from hiPSC-CMs that have not only generated physiologically relevant CVs in excess of 30 cm/s (Yang et al., 2014), but generated velocities close to 50 cm/s with these cells (Figure 8B). Whether this increase in structure functionality is a function of cell-cell communication and paracrine signaling, a function of increased culturing time, or a combination of these factors remains to be seen. It has been shown, however, that cFBs do affect the maturation of hiPSC-CMs through secretion of growth-related cytokines when co-cultured (Ieda et al., 2009). These data suggest that superior electrophysiological function of the current LbL approach may be in part caused by the addition of the cFB layer.

It should be noted that, even though this 3D *in vitro* study enhanced our knowledge-base of thick, engineered cardiac tissue structures and some of their electrophysiological capabilities, further studies are required to better understand the mechanical aspects of the system. In order to better understand, model future system and avoid mechanical mismatch when implanting these structures, their viscoelastic properties need to be determined and related to the dynamic properties of the ECM (Little and Wead, 1971; Tsaturyan et al., 1984; Miller and Wong, 2000; Wang et al., 2010).

## Conclusion

Here we have shown that a modular fabrication method, like LbL assembly, can be utilized to produce thick (∼2.12 mm), viable engineered cardiac tissues from hiPSCs. The incorporation of cardiac FBs into the LbL assembly method allowed for superior, previously unobtainable CVs (>30 cm/s) along with a lack in arrhythmogenic potential, with tissues having the ability to be paced to 5 Hz (200 ms). *In vitro* characterization showed engineered tissue structures resembling those of native cardiac tissue along with minimal necrosis, even after 4 weeks in culture. Considering that the tissues engineered for this study have not undergone any form of maturation, nor have they been exposed to any external stimuli, suggests that their performance capabilities could be enhanced even further.

Future work will include the mechanical testing of the engineered cardiac tissues to determine viscoelastic properties to ultimately prevent mechanical mismatch between the engineered tissues and future host. *In vivo* studies in a large animal model will also commence in order to assess the potential clinical application of these larger, thicker, engineered cardiac tissues in preventing LV dilatation of hearts with postinfarction LV remodeling.

## Data Availability Statement

The raw data generated for this study are available on request to the corresponding author.

## Author Contributions

DP designed the LbL fabrication technique, designed the experiments, performed histology and imaging, sample preparation, and wrote the manuscript. AK-K performed the cell differentiation, sample staining, and imaging as well as RNA analysis. XL performed the cell differentiation, sample staining, and imaging. VF performed the optical mapping. JB reviewed the manuscript. TK supplied cFBs and reviewed the manuscript. JZ provided the project leadership, funding acquisition, method development, and manuscript revisions. All authors contributed to the article and approved the submitted version.

## Conflict of Interest

TK is a consultant for Fujifilm CDI. The remaining authors declare that the research was conducted in the absence of any commercial or financial relationships that could be construed as a potential conflict of interest.
