# Supplementary material for: Layer-By-Layer Fabrication of Large and Thick Human Cardiac Muscle Patch Constructs With Superior Electrophysiological Properties
Source: Front Cell Dev Biol. 2021 Apr 16;9:670504. doi: 10.3389/fcell.2021.670504 (PMC8086556; doi:10.3389/fcell.2021.670504)
Supplement: Supplementary file 1 [file Data_Sheet_1.PDF]

1.1 Supplementary Figures

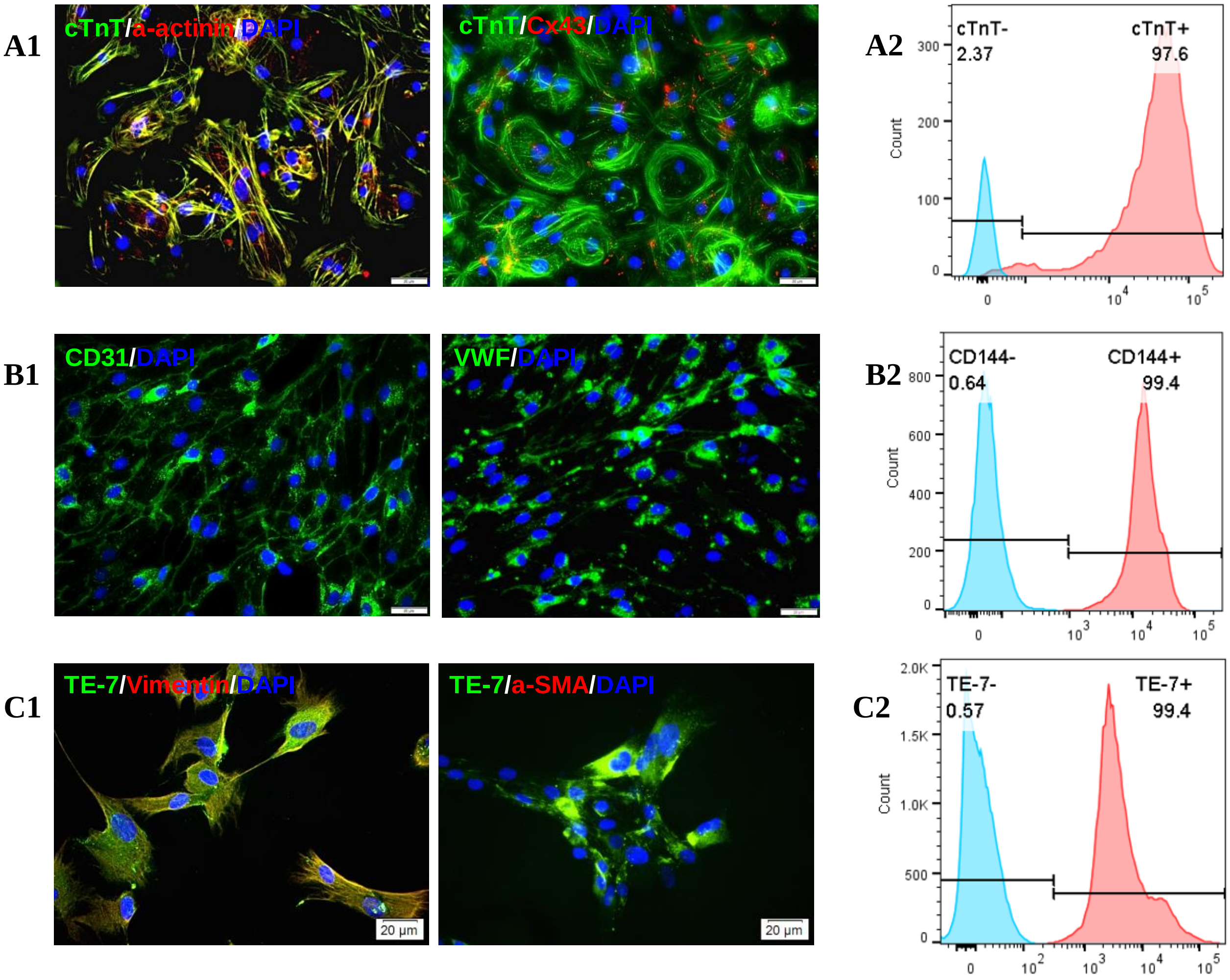

Supplementary Figure 1: Characterization data confirming differentiation and purification of iCMs (A), iECs (B) and cFBs (C) using immunofluorescent staining (1) and flow cytometry (2) analysis.

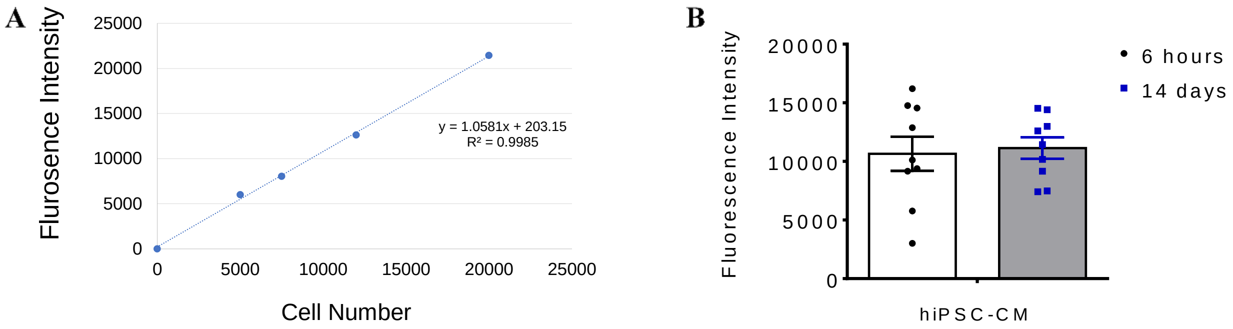

Supplementary Figure 2: Fluorescence-based cell proliferation assay showing the (A) standard curve and (B) the resulting measurements at 6 hours of hiPSC-CMs culture in a 96-well plate (10,000 cells/well) as well as measurements after 14 days of culture. $p = 0.783$, $n = 9$

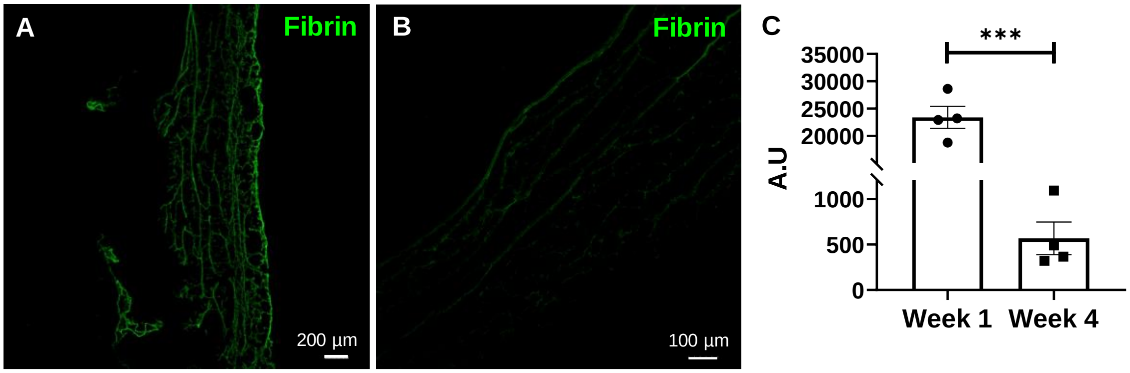

Supplementary Figure 3: Representative confocal images of fibrin degradation over a 4-week period, with (A) showing fibrin expression levels at week 1, (B) showing fibrin expression levels at week 2, and (C) showing quantification of relative expression levels at each time point (n = 4, ***p < 0.001)

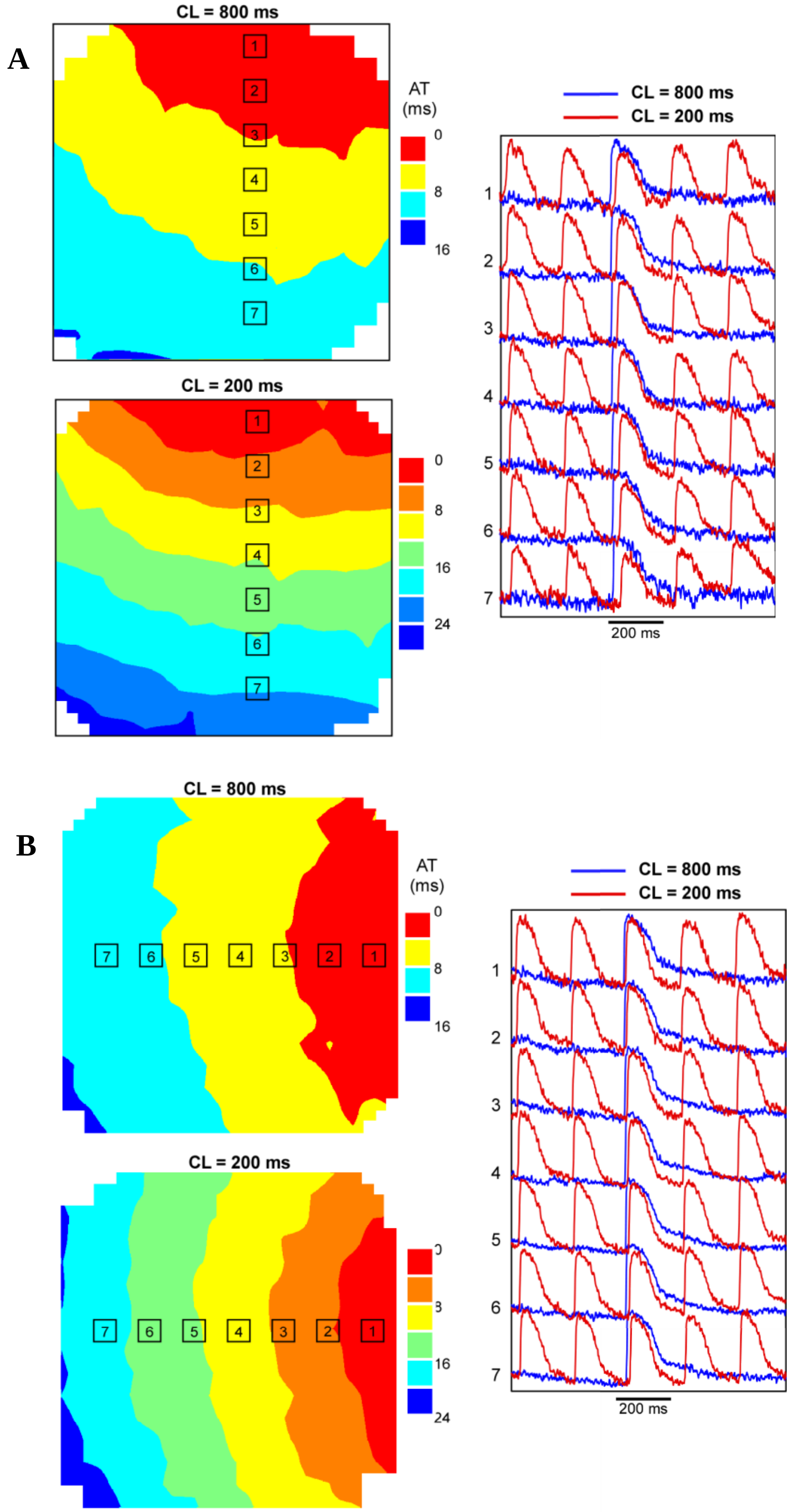

Supplementary Figure 4: Representative heatmaps of engineered tissue conduction capabilities, with representative signal propagation heatmap as well as pacing at both 200 ms (red) and 800 ms (blue) for (A) week 2, and (B) week 4 samples

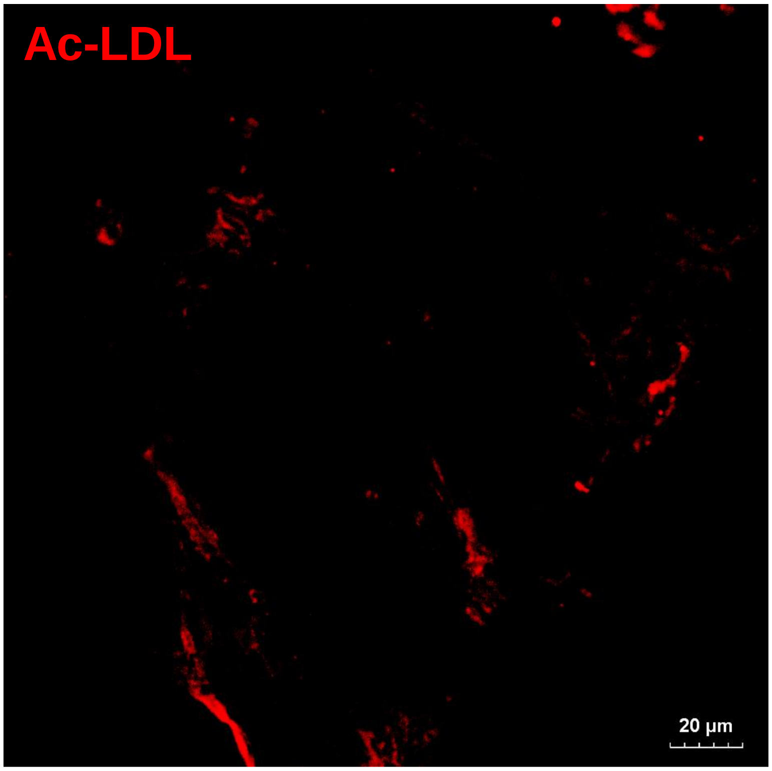

Supplementary Figure 5: Ac-LDL assay showing vessel-like structures forming throughout the thick LbL engineered tissue after 10 days in culture.

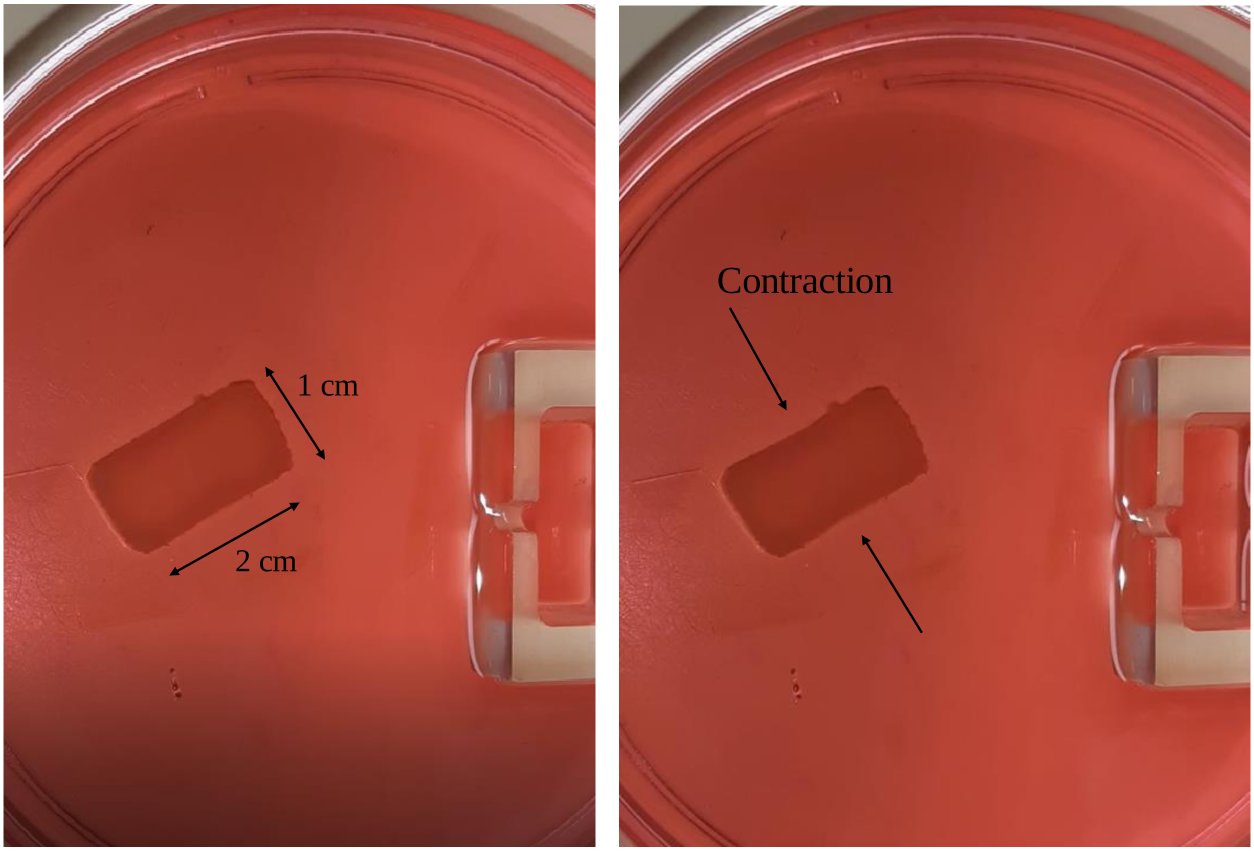

Supplementary Figure 6: Image of the thick 1x2 cm^2 thick LbL engineered cardiac tissue outside of its frame

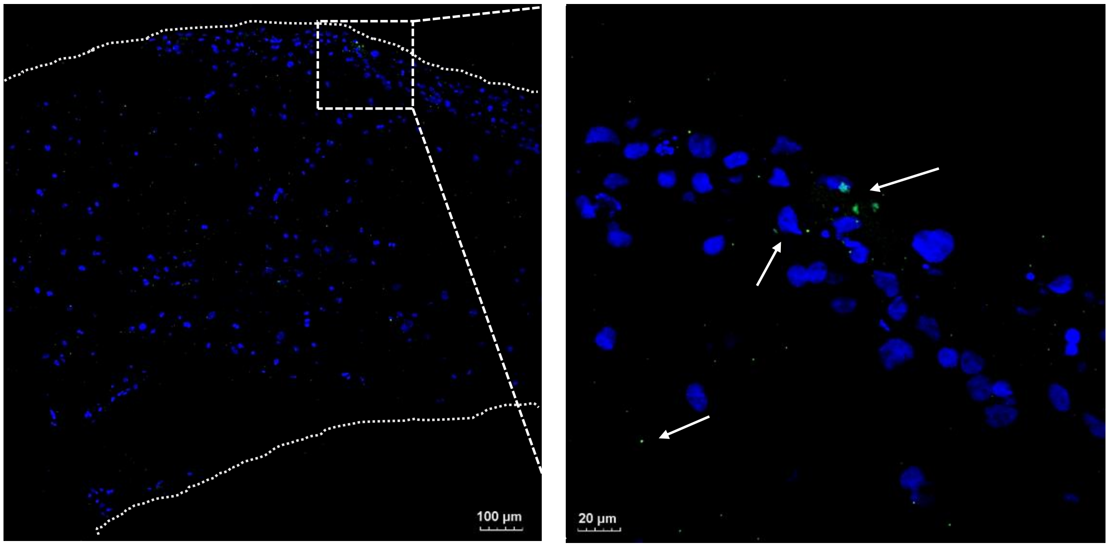

Supplementary Figure 7: Representative confocal micrograph of engineered LbL cardiac tissue displaying necrotic cells identified by the necrosis marker phosphorylated MLKL (Ser358, pMLKL) at week 2 of culture. Border indicated with short dashed line
