# Supplementary material for: Layer-By-Layer Fabrication of Large and Thick Human Cardiac Muscle Patch Constructs With Superior Electrophysiological Properties
Source: Front Cell Dev Biol. 2021 Apr 16;9:670504. doi: 10.3389/fcell.2021.670504 (PMC8086556; doi:10.3389/fcell.2021.670504)
Supplement: Supplementary file 2 [file Table_1.DOCX]

Supplementary Material

# Supplementary Materials and Methods

## Flow cytometry analysis

Flow cytometry analysis was performed as described previously (Gao et al. 2018;Zhang et al. 2019). Briefly, cells were dissociated using 0.25 % trypsin and resuspended as single cells, permeabilized in 0.1 % Triton X-100 at 4 °C for 10 min, then incubated with primary and secondary antibodies for 30 min at 4 °C with 3 x 5 min wash in between. Finally, cells were resuspended in 2% fetal bovine serum/phosphate-buffered saline (FBS/PBS) containing 5 μL of propidium iodide (10 μg/mL) and evaluated with a FACS Aria instrument (BD Biosciences, USA). Antibodies used, along with dilutions, are listed Supplementary Table 1.

## Cardiomyocyte proliferation assay

Cardiomyocyte proliferation was tested using a proliferation assay from CyQUANT Direct Cell Proliferation Assay Kit (Invitrogen, Cat# C35011). Briefly, CM proliferation was determined via green fluorescent nucleic acid staining of the nucleus, after which the fluorescence intensity of the readout was obtained 60 min after reagent addition via microplate reader in a 96-well plate (10 000 cells/well). Based on the results, there was no significant difference between the proliferation of 6-hour-old and 14-day-old hiPSC-CMs (see Supplementary Figure 2). Student t-test was performed, p = 0.783 (n = 9).

## Image analyses

All image quantification analyses were performed with ImageJ. Where indicated, arbitrary units (A.U) are representative of a pixel count and intensity for each sample. All samples were stained and imaged under similar conditions to allow for a comparative study.

## Tube-formation assay

Tube-formation in thick LbL engineered cardiac tissue was analyzed by incubating tissue in their optimized DMEM media with 0.05% FBS for 2 hours at 37 °C. Following incubation, media was removed and replaced with normal optimized DMEM media containing 2% FBS + Dil-Ac-LDL (10 µg/mL final concentration, Thermo Fisher # L35354) and incubated for 4 hours at 37 °C. Tube formation was then visualized using confocal microscope.

# Supplementary Figures and Tables

## Supplementary Tables

Supplementary Table 1: Antibodies used for flow cytometry, FACS, and immunofluorescent staining.

| Antibody Name | Application | Company | Catalog Number |
| --- | --- | --- | --- |
| Alexa Fluor® 647 Mouse Anti-Human CD31 | FACS | BD Biosciences | 561654 |
| Cardiac Troponin T Monoclonal Antibody (13-11) | Flow Analysis | Invitrogen | MA5-12960 |
| Anti-alpha smooth muscle Actin antibody | Immunofluorescent Staining | Abcam | 21027 |
| Rabbit Anti-Cardiac Troponin T antibody [EPR3695] | Immunofluorescent Staining | Abcam | 91605 |
| Mouse Anti-Cardiac Troponin T antibody [1F11] | Immunofluorescent Staining | Abcam | 10214 |
| Goat anti-Mouse IgG Secondary Antibody, Alexa Fluor 555 | Immunofluorescent Staining | Invitrogen | A32727 |
| Donkey anti-Mouse IgG Secondary Antibody, Alexa Fluor 488 | Immunofluorescent Staining | Invitrogen | A21202 |
| Donkey anti-Rabbit IgG Secondary Antibody, Alexa Fluor 488 | Immunofluorescent Staining | Invitrogen | A21206 |
| Donkey anti-Rabbit IgG Secondary Antibody, Alexa Fluor 555 | Immunofluorescent Staining | Invitrogen | A31572 |
| Rabbit Anti-Collagen I antibody | Immunofluorescent Staining | Abcam | 34710 |
| Mouse Anti-CD31 antibody [JC/70A] | Immunofluorescent Staining | Abcam | 9498 |
| Rabbit Anti-Fibrinogen beta chain antibody | Immunofluorescent Staining | Abcam | 137830 |
| Rabbit Anti-Collagen III antibody | Immunofluorescent Staining | Abcam | 7778 |
| Rabbit Anti-Fibronectin antibody | Immunofluorescent Staining | Abcam | 2413 |
| Rabbit Anti-Collagen IV antibody | Immunofluorescent Staining | Abcam | 6586 |
| Rabbit Anti-Laminin antibody | Immunofluorescent Staining | Abcam | 11575 |
| Rabbit Anti-VE Cadherin | Immunofluorescent Staining | Abcam | 33168 |
| Mouse Anti-VWF Antibody (F8/86) | Immunofluorescent Staining | Santa Cruz Biotech | 53466 |
| Rabbit Anti-N-Cadherin | Immunofluorescent Staining | Abcam | 18203 |
| Anti-Alpha Actinin | Immunofluorescent Staining | Sigma | A7811 |
| Anti-Connexin 43 / GJA1 antibody - Intercellular Junction Marker | Immunofluorescent Staining | Abcam | 11370 |
| Mouse Anti-Ryanodine Receptor antibody [C3-33] | Immunofluorescent Staining | Abcam | 2827 |
| JPH2 Polyclonal Antibody | Immunofluorescent Staining | Thermo Fisher | 40-5300 |
| Recombinant Anti-Cardiac Troponin T antibody | FACS | Abcam | 91605 |
| Mouse anti-Human CD144 | FACS | BD Biosciences | 560410 |
| Anti-Fibroblasts Antibody | FACS | Millipore Sigma | CBL271 |

Supplementary Table 2: Formulation of cardiac fibroblast differentiation basal medium (CFBM)

| **Components** | **Final concentration** |
| --- | --- |
| DMEM, high glucose (4.5 g/L) | basal medium |
| HLL Supplement: HSA (human serum albumin), linoleic acid and lecithin | HSA: 500 µg/mL  Linoleic Acid: 0.6 µM  Lecithin: 0.6 µg/mL |
| Ascorbic Acid | 50 μg/mL |
| GlutaMAX | 7.5 mM |
| Hydrocortisone Hemisuccinate | 1.0 μg/mL |
| rh Insulin | 5 μg/mL |

Supplementary Table 3: Primers used for RNA analyses

| **Target Gene** | **Forward Primer** | **Reverse Primer** |
| --- | --- | --- |
| cTnT | TTCACCAAAGATCTGCTCCTCGCT | TTATTACTGGTGTGGAGTGGGTGTGG |
| CD31 | TCAGACGTGCAGTACACGGA | GGGAGCCTTCCGTTCTAGAGT |
| Alpha SMA | TATCCCCGGGACTAAGACGG | CACCATCACCCCCTGATGTC |
| Periostin | AGGTCACCAAGGTCACCAAATTC | CTCACGGGTGTGTCTCCCTG |
| Vimentin | CCTCCGGGAGAAATTGCAGG | TCAAGGTCAAGACGTGCCAG |
| FAP | AGGGATGGTCATTGCCTTGG | ATCCTCCATAGGACCAGCCC |
| Alpha MHC | CTCCGTGAAGGGATAACCAGG | TTCACAGTCACCGTCTTCCC |
| Beta MHC | ACCAACCTGTCCAAGTTCCG | TCATTCAAGCCCTTCGTGCC |
| MLC2a | ggagttcaaagaagccttcagc | AAAGAGCGTGAGGAAGACGG |
| MLC2v | ACATCATCACCCACGGAGAAGAGA | ATTGGAACATGGCCTCTGGATGGA |
| Collagen 1 | TGACGAGACCAAGAACTGCC | GCACCATCATTTCCACGAGC |
| Collagen 3 | GGATGGTTGCACGAAACACAC | GGTAGTCTCACAGCCTTGCG |
| Collagen 4 | GGCAGATTCGGACCACTAGG | GCGTCTGTGGCAATACTAGC |
| Fibronectin | TCGTGCTTTGACCCCTACAC | CGGGAATCTTCTCTGTCAGCC |
| Laminin | ACTTGAGTATGAAAGCAAGGCCAG | GGAGAGCTCCACAAAACCAGG |
| Elastin | GTGTCTGCAGGTGCGGTG | CTGGGTATACACCTGGCAGC |
| SERCA | TCACCTGTGAGAATTGACTGG | AGAAAGAGTGTGCAGCGGAT |
| RyR2 | TTGGAAGTGGACTCCAAGAAA | CGAAGACGAGATCCAGTTCC |
| CACNA1C | TGATTCCAACGCCACCAATTC | GAGGAGTCCATAGGCGATTACT |
| JPH2 | CCAAGTATGAGGGCACCTGG | GCCTTGGTACGTCCCTCCAT |
| Cx43 | GGTGACTGGAGCGCCTTAG | GCGCACATGAGAGATTGGGA |
| Tbx20 | ATTCCTATGCACGCTCACCC | TGTTGTAAAGGCTGACCCTCG |
